# Artery of Percheron Infarct: A Rare Case Presentation and Recovery

**DOI:** 10.7759/cureus.44033

**Published:** 2023-08-24

**Authors:** Ronald Lott, Xavier Zonna, Aneesha Nadukudiyil Jose, Justin Shin, Maximilian Roemer, Asad Nasir

**Affiliations:** 1 Internal Medicine, Lake Erie College of Osteopathic Medicine, Erie, USA; 2 Internal Medicine, Lake Erie College of Osteopathic Medicine, Elmira, USA; 3 Internal Medicine, Arnot Ogden Medical Center, Elmira, USA; 4 Critical Care, Arnot Ogden Medical Center, Elmira, USA

**Keywords:** midbrain infarction, comatose, cerebrovascular stroke, bilateral thalami infarction, artery of percheron infract

## Abstract

The artery of Percheron (AOP) is a congenital anatomical irregularity of the cerebrovasculature responsible for perfusing the thalamus and midbrain. These defects account for a small percent of all ischemic strokes and present with widely variable symptomatology, ranging from confusion to coma. We present a case of an acute AOP infarct and recovery in a 68-year-old male with atrial fibrillation without anticoagulation. It is our hope that this case report serves to alert diagnosticians to the presence of AOP infarcts, the wide clinical presentation, and the prognosis of this rare and critical type of ischemic stroke.

## Introduction

The artery of Percheron (AOP) represents a rare anatomical variant responsible for bilateral thalamic and midbrain perfusion. The prevalence of AOP is estimated to be 4-12% in the general population [1] and is a rare etiology of stroke accounting only for 0.1-2% of ischemic strokes [2]. There are four common patterns to AOP infarctions. These include bilateral paramedian thalamic with midbrain (43%), bilateral paramedian thalamic without midbrain (38%), bilateral paramedian thalamic with anterior thalamus and midbrain (14%), and bilateral paramedian thalamic with anterior thalamus without midbrain (5%) [1].

The clinical presentation of an AOP infarct is widely variable based on what areas of the thalamus are involved. For example, if the mediodorsal nucleus of the thalamus is involved, patients may present with memory impairment, behavioral changes, psychosis, aphasia, and dysarthria. Alternatively, if the rostral midbrain is involved with the AOP, the patient may present with oculomotor abnormalities [3]. Patients may also present with coma as the thalamus functions to maintain consciousness [4]. The wide variability of this condition necessitates a high index of suspicion for diagnosis. Bilateral thalamic lesions can also have variable etiologies, including infectious, malignant, inflammatory, or vascular causes [1]. This variability in presentation and lack of prevalence in the general population leads to significant difficulty in clinically diagnosing these rare infarctions, especially without MRI [5].

This case report details a unique presentation of an AOP infarction causing somnolence, dysarthria, and bilateral motor abnormalities believed to be in the setting of paroxysmal atrial fibrillation without anticoagulation due to patient noncompliance. The goal of this report is to add to the paucity of literature describing an AOP infarct and to educate diagnosticians on the variability of this infarct, as well as the difficulty in diagnosis with conventional imaging.

## Case presentation

We present a case of a 68-year-old male who presented to the emergency room with a complaint of new-onset altered mental status and falls. Our patient has a notable history of hypertension, hyperlipidemia, diabetes, and paroxysmal atrial fibrillation/flutter rate controlled on sotalol 150 mg outpatient without anticoagulation due to patient noncompliance. The patient was oriented to time, person, and place on arrival but was noted to be very somnolent. Upon questioning, the patient’s wife reports that, earlier in the day, the patient had been highly fatigued and suffered multiple falls from imbalance while walking to the bathroom. However, the patient did not immediately call emergency medical services (EMS). After some time, EMS was contacted and transported the patient to the hospital. It was determined during the initial history that, if the patient had a stroke, he was outside the window for thrombolysis.

**Figure 1:**
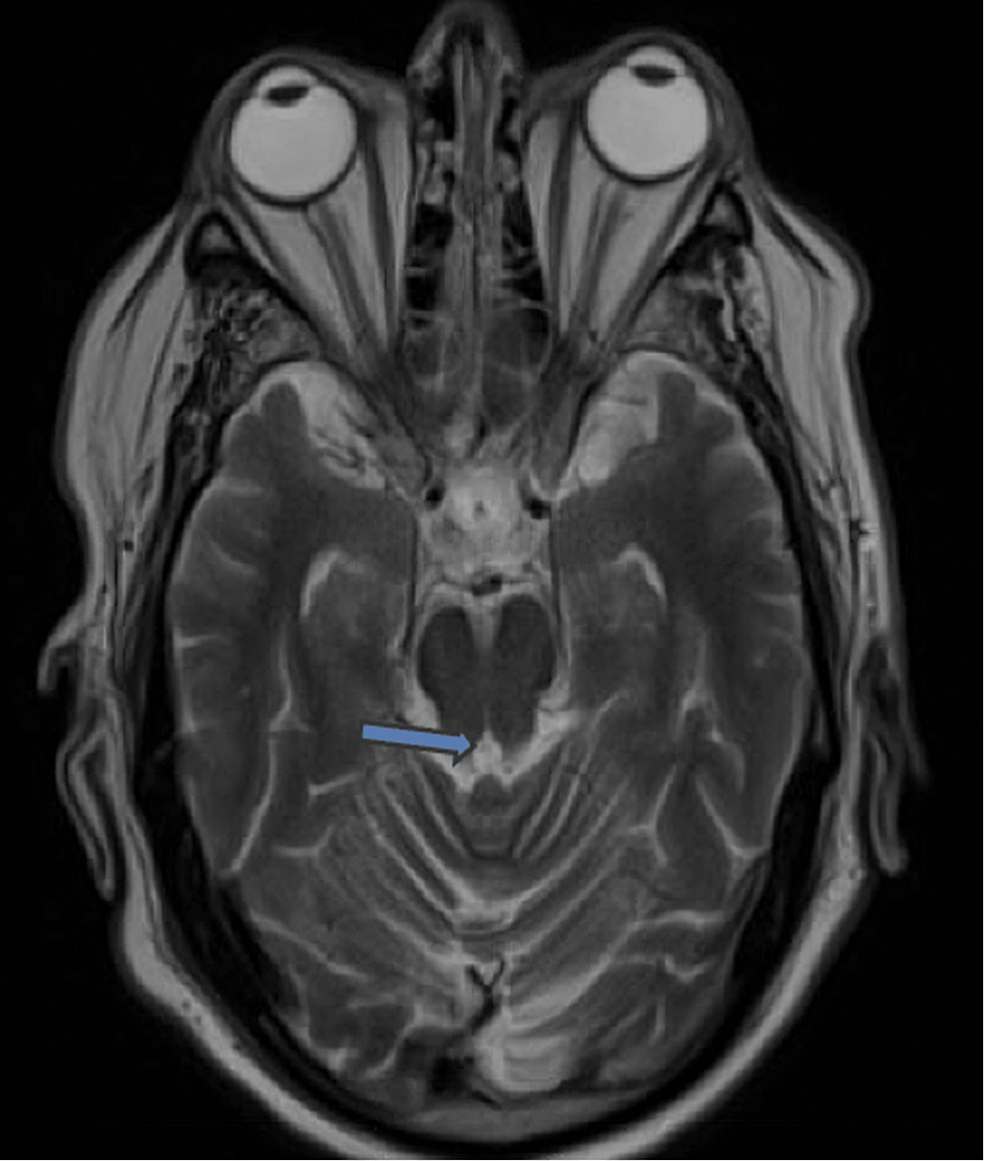
T2 blade axial imaging demonstrating an increased signal in the medial midbrain, noted by the blue arrow.

**Figure 2:**
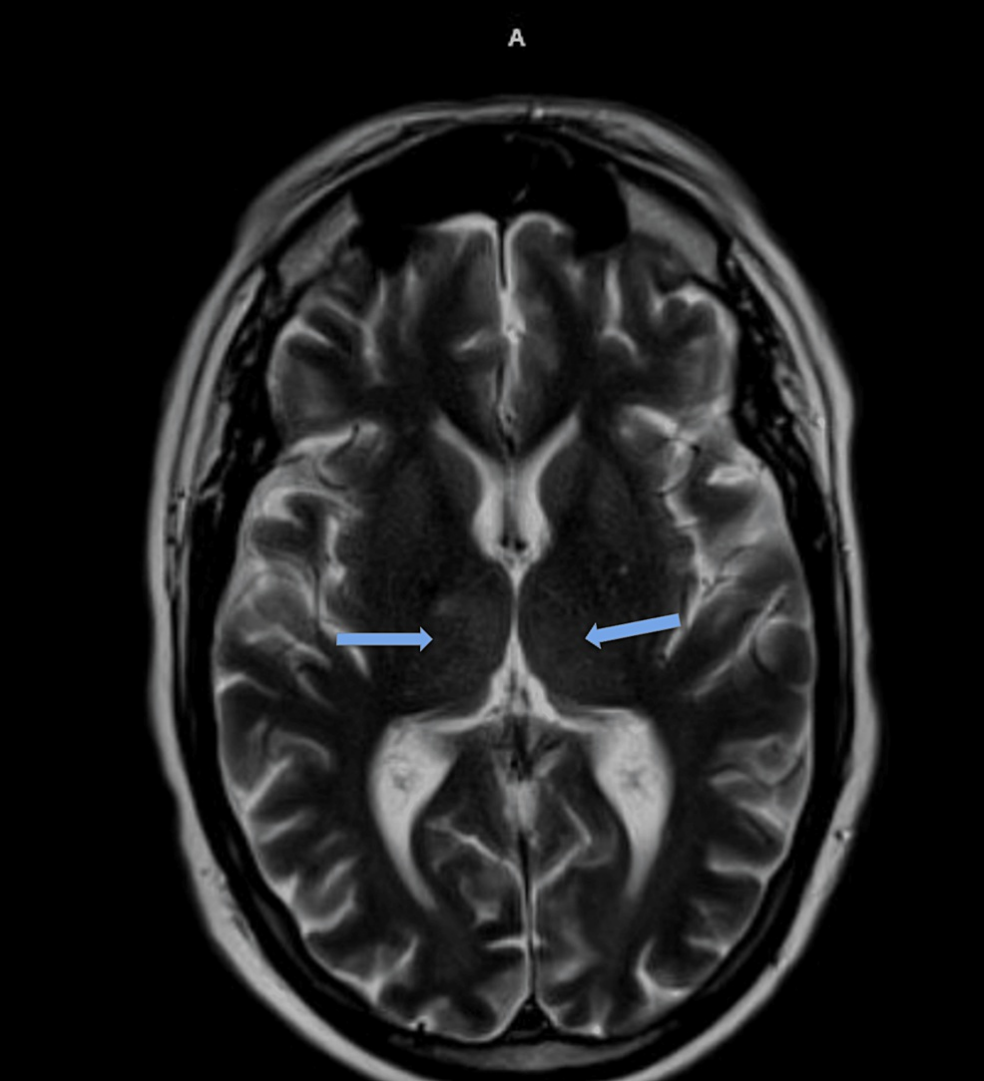
T2 flair axial imaging with an increased uptake in the thalami, bilaterally noted by the blue arrows.

While in the emergency department, the patient was oriented to time, person, and place but was found to be very somnolent. He was noted to have fixed, dilated pupils on the exam. A CT head and CTA/CVA were ordered to rule out stroke or brain bleeding in light of the patient’s somnolence and history of falls. Upon returning from the CT scan, the patient was found to be more somnolent and unresponsive to sternal rubs and commands. At this time, the patient was intubated with an endotracheal tube for airway protection and placed on a ventilator at a FiO2 of 30%, a tidal volume of 500, a respiratory rate of 16, and a PEEP of 5. Simultaneously, the patient was noted to have a blood pressure of 212/150. Thus, he was started on a nicardipine drip for blood pressure control with a goal pressure of less than 160 systolic. Due to the patient’s worsening status, the critical care team was contacted, and the patient was admitted to the ICU. CT head and CTA/CVA from admission demonstrated no acute intracranial hemorrhage or significant vessel obstruction, no mass, no aneurysm, and no dural venous thrombosis with patent carotid arteries. However, a circle of Willis variant was noted at this time. After initial stabilization, an MRI of the brain with and without contrast was ordered for a suspected cerebrovascular accident. MRI revealed infarcts of the medial aspect of the thalami bilaterally, as well as the upper medial midbrain with a suspected age of infarct between six and 16 hours suspicious of an infarct involving the AOP (Figures 1-2). Consequently, a neurology consult was ordered, as well as an echocardiogram with bubble study, hemoglobin A1c, and lipid panel.

Overnight, the patient self-extubated, but, when examined, remained unresponsive to voice commands. He was placed on vapotherm 30 L/min to maintain his respiratory status. On hospital day two, the patient remained minimally oriented, without the ability to follow commands or safely consume liquids or food. The patient was also noted to be in sinus rhythm at this time, despite a reported history of atrial fibrillation/flutter. However, there are documented instances of atrial fibrillation on telemetry and exams during this hospital stay.

On hospital day three, the patient continued to remain unresponsive to commands despite no sedation. He was noted to have bilateral flailing of his arms without purposeful movement. He was also noted to be muttering unintelligible words on the exam. His oxygen requirements also decreased at this time, as he required only 2 L of nasal cannula O2 for adequate saturation. A repeat head CT was completed to evaluate for hemorrhagic conversion. However, the scan demonstrated an evolving ischemic infarct without hemorrhagic conversion. The patient was also started on aspirin therapy per neurology’s recommendations.

By hospital day four, the patient remained unresponsive to commands but did mutter unintelligible words in response to voice. His bilateral flailing of the extremities continued on hospital day four. His activity level was increased, although unpurposeful, and a sitter was subsequently ordered to protect the patient.

From hospital day five onward, the patient’s neurological status improved tremendously. During the exam on day five, the patient was oriented to person and place. By day six, the patient was able to follow commands to smile, move his arms, move his legs, and respond to increasingly complex questions in a sensical manner. Although his speech remained dysarthric, the team could understand his responses with some effort. On hospital day seven, the patient could ambulate for the first time with two assistants. The patient’s speech also improved. The patient continued to improve neurologically during his stay and was evaluated by PT/OT and speech therapy. By hospital day 10, the patient was noted to be near his cognitive baseline by family members with only intermittent periods of confusion requiring orientation and occasional slow responses to questioning. He also has some continued left-sided weakness and facial droop.

As this stroke is thought to be cardioembolic in origin due to a history of atrial fibrillation without anticoagulation, the patient was placed on apixaban 5 mg twice a day for anticoagulation therapy. Although the patient will need neurological rehabilitation and close outpatient follow-up from a multidisciplinary team, the patient cognitively recovered quickly despite a bilateral AOP infarct involving both the midbrain and thalamus.

## Discussion

A recently published review in neurology found that, in an eight-year review of one institution’s neuroimaging database, only 14 confirmed cases of AOP were found. Of these, 57% were female, with the median age of presentation at 67.5 years. These patients most commonly presented with elevated systolic blood pressure >140 (78.5%) and decreased level of consciousness (42.8%), just as the patient in our case. Additionally, only one patient (7.1%) was eligible for and received thrombolysis as the median arrival time to the emergency department was 14.1 hours [6]. Although AOP infarcts are known for causing long-term deficits, it is evident that a majority of cases present late in the clinical course diminishing the chance of treatment with thrombolysis [6]. Endovascular treatment is often not possible as well due to the small diameter of the vessel [7].

Not only do AOP infarctions typically present outside of the thrombolysis window, but the large variety of symptoms can make clinical recognition of this condition difficult. Anatomically, the AOP represents a single artery that originates from the posterior cerebral artery and bifurcates to perfuse the bilateral thalami [8]. Typically, the posterior cerebral artery on each side has a paramedian thalamic artery that supplies blood flow to its respective thalami. Consequently, in individuals with an AOP, ischemia to the AOP may result in bilateral deficits involving the thalami and midbrain. Multiple stroke patterns involving the AOP have been described including bilateral paramedian thalamic, paramedian and polar thamalic, and paramedian thalamic and mesencephalic [1]. The common outcomes of a bilateral paramedian thalamic stroke, as demonstrated in our patient, include the triad of altered mental status, vertical gaze palsy, and memory impairment [1]. A 2014 study also found that, in 15 patients with AOP infarctions, mental status changes and oculomotor disturbances were the two most common findings [9]. These findings were corroborated in a separate stroke registry study that found 10 patients with AOP infarcts to have behavioral, cognitive, and consciousness disturbances at the onset of stroke [10]. However, studies report variations in symptoms in a spectrum of seven patterns as follows: mental status changes, aphasia, behavior/memory impairment, ocular movement abnormalities, cerebellar signs, motor deficits, and nonspecific signs, such as seizures, hyperthermia, and hypersomnia [8,11]. Symptoms such as decreased consciousness or coma are not common during stroke, and clinicians should consider AOP involvement in these unusual cases [4].

In addition to variable clinical presentations, diagnosing AOP infarcts even with radiographic imaging is a challenge. MRI is considered the modality of choice but can be negative on initial readings, and repeat imaging may be necessary to successfully diagnose this pathology [5]. CT angiography, while usually helpful for localizing vascular blockages, may be falsely negative for many patients with AOP infarctions [8]. This is due to the fact that the absence of the AOP does not alarm the reader due to its rarity in the general population [5]. A 2021 case report described a patient presenting within 4.5 hours but not being diagnosed within the thrombolytic time due to nondiagnostic CT of the head and CT angiography, as well as a clinical presentation of altered mental status and fluctuating levels of consciousness [5]. These delays can have major implications for the future health of patients, and future research is needed to determine the most accurate means of quickly and effectively recognizing these rare strokes. Clinicians should be aware of falsely negative CTA as a potential clue to consider AOP in patients with stroke symptoms but initially negative CTA. Serial MRI imaging should also be performed when an AOP infarction is suspected.

Further research is also needed to determine the long-term prognosis of patients with AOP infarctions. The prognosis can vary widely based on the type of infarction and the affected regions. Smaller-scale trials have determined that the prognosis is generally favorable unless the patient’s infarction has midbrain involvement [9]. However, poor outcomes can also be associated without midbrain involvement. A case series found that 26% of patients had complete recovery, and all infarctions were bilateral paramedian thalamus without midbrain involvement [1]. Further research is needed to clarify the true long-term prognosis of all four types of AOP infarctions. For patients with AOP infarctions that contain thalamic and midbrain involvement, there can be persistent long-term deficits in executive function and processing speed, as well as both working and executive memory one year after the infarction [10].

## Conclusions

Our case demonstrates a rare presentation of an AOP infarction affecting the bilateral midbrain and thalami. These infarctions are highly variable in patient presentation and often have debilitating effects on the patient. This case is unusual due to the patient’s expeditious return to near cognitive baseline even with high-risk involvement of the midbrain on MRI. The patient progressed from a near comatose state to being alert, oriented, and engaged in conversation by day 10 of hospital admission. It is our hope that this case presentation alerts clinicians to these rare infarctions, their varying presentation, and their typical clinical progression.
